# Auditory feedback blocks memory benefits of cueing during sleep

**DOI:** 10.1038/ncomms9729

**Published:** 2015-10-28

**Authors:** Thomas Schreiner, Mick Lehmann, Björn Rasch

**Affiliations:** 1 Department of Psychology, University of Zurich, 8050 Zurich, Switzerland; 2 Department of Psychology, University of Fribourg, 1701 Fribourg, Switzerland; 3 Psychiatric University Hospital Zurich, Clinic of Affective Disorders and General Psychiatry, 8032 Zurich, Switzerland; 4 Zurich Center for Interdisciplinary Sleep Research (ZiS), 8091 Zurich, Switzerland

## Abstract

It is now widely accepted that re-exposure to memory cues during sleep reactivates memories and can improve later recall. However, the underlying mechanisms are still unknown. As reactivation during wakefulness renders memories sensitive to updating, it remains an intriguing question whether reactivated memories during sleep also become susceptible to incorporating further information after the cue. Here we show that the memory benefits of cueing Dutch vocabulary during sleep are in fact completely blocked when memory cues are directly followed by either correct or conflicting auditory feedback, or a pure tone. In addition, immediate (but not delayed) auditory stimulation abolishes the characteristic increases in oscillatory theta and spindle activity typically associated with successful reactivation during sleep as revealed by high-density electroencephalography. We conclude that plastic processes associated with theta and spindle oscillations occurring during a sensitive period immediately after the cue are necessary for stabilizing reactivated memory traces during sleep.

## Abstract (web summary)

Exposure to memory cues during sleep improves subsequent memory recall. Here the authors demonstrate that presenting an additional auditory stimulus during a critical time window following the memory cue abolishes the memory benefit of cueing and its oscillatory correlates during sleep in humans.

## Graphical abstract

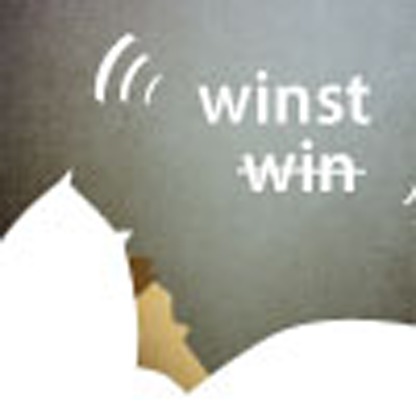

Reactivation has a crucial role in the maintenance of long-term memories. Memory reactivation underlies almost all active recall processes, intentionally bringing the memorized information from memory to the focus of our attention^1^,^2^,^3^. Repeated active and successful recall attempts are known to be particularly effective in further strengthening the memory trace for the long term^4^ and might represent a core mechanism for forming abstract and semantic long-term memories (for example, multiple trace theory^5^). Memories can also be reactivated by exposure to associated memory cues, thereby facilitating intended memory recall^6^ or inducing unintentional memory retrieval^7^. The consequence of reactivation by cueing on later memory strength is twofold: if cued reactivation is immediately followed by interfering inputs, the reactivated memory might be destabilized and even forgotten as predicted by reconsolidation theory^8^. In contrast, presentation of a cue followed by correct feedback (for example, the associated item in paired-associated learning) mostly reinforces the to-be-learned memory association and leads to a strengthening of the memory trace^9^. Thus, the fate of a memory after its reactivation appears to be strongly dependent on the degree of overlap between the expected and the true input after reactivation, that is, the resulting prediction error^10^,^11^,^12^.

In addition to waking, reactivation is also assumed to underlie the beneficial role of sleep for memory^13^. According to the active system consolidation hypothesis, memories are spontaneously reactivated during Non rapid-eye movement (NREM) sleep, which strengthens and integrates newly acquired memories into cortical knowledge networks^14^,^15^,^16^. Cortical slow oscillations (<1 Hz) coordinate these reactivation processes as a time-giving pace maker, and drive repeated reactivations of memory representations in the hippocampus together with sharp wave ripples and thalamo-cortical sleep spindles. Formation of these spindle-ripple events is assumed to represent a core mechanism to enable the redistribution of reactivated hippocampal memory information to neocortical long-term stores, leading to a subsequent stabilization and strengthening of the reactivated memory^14^.

Various recent studies have established a causal role of reactivation for consolidation processes during sleep by showing that cueing memories during NREM sleep improves later memory recall^17^,^18^,^19^,^20^. In these studies, memory cues (that is, olfactory, auditory) are repeatedly presented during sleep to reactivate the associated memory content, leading to a strengthening of the memory tested after sleep. However, the processes necessary for stabilizing memory representations after their reactivation during sleep are not well understood. In particular, it is still unknown whether additional input after cueing during sleep might improve or interfere with ongoing stabilization processes, and whether these effects are dependent on the degree of overlap between expected and true input as during wakefulness.

Here, we aimed at identifying the critical processes supporting a strengthening of memories upon their reactivation during sleep. To test the role of a reactivation-associated prediction error during sleep, we presented correct versus false auditory feedback after reactivation by cueing and examined the underlying oscillatory correlates in healthy human subjects. Recently, we could show that foreign vocabulary cues are capable of inducing reactivation processes during sleep and thereby boosting memory performance^21^. German-speaking participants had to learn Dutch–German word pairs. During subsequent NREM sleep, half of the Dutch words were replayed via loudspeaker leading to enhanced memory for the German translation of the cued words. We demonstrated that strengthening of memories by cueing during sleep was associated with a temporary increase in theta as well as spindle activity after re-exposure to verbal memory cues.

Here, we basically used the same paradigm, but additionally introduced auditory feedback (that is, correct or false German translations) after cue presentation during NREM sleep for some Dutch words (cued words with feedback). In the following we refer to the Dutch words as cues and the German words as feedback, which was correct or false with respect to the initially learned association between the Dutch word and the German translation. False feedback was created by randomly intermixing Dutch–German pairs from a subset of the original learning list. We hypothesized that the fate of a reactivated memory during sleep depends on the degree of prediction error after its reactivation: we expected that presentation of correct feedback after cueing leads to an additional improvement in later memory recall of the German translation, whereas, exposure to interfering false feedback should result in increased forgetting of the Dutch vocabulary after sleep. In contrast to our expectation, presenting feedback immediately after the memory cue completely blocked the memory benefits of cueing during sleep, independent of its content. A similar blockade was observed when using a tone instead of verbal feedback. In contrast, inserting a time delay between cue and verbal feedback restored the memory benefits of cueing. On a neural basis, immediate (but not delayed) auditory stimulation abolished the characteristic increases in oscillatory theta and spindle activity typically associated with successful reactivation during sleep^21^ and/or sleep-dependent memory consolidation^22^,^23^. We conclude that plastic processes associated with theta and spindle oscillations occurring during a sensitive period immediately after the cue are necessary for stabilizing reactivated memory traces during sleep.

## Results

**Figure 1:**
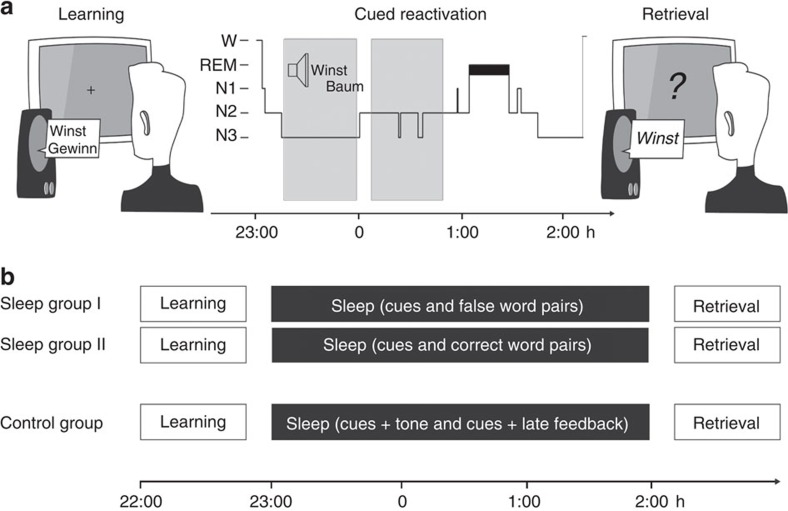
Experimental procedure. (**a**,**b**) After studying 120 Dutch–German word pairs in the evening, participants slept for 3 h. During the retention interval 80 Dutch words (40 cued, 40 cued+feedback) were repeatedly presented in sleep groups I and II. The type of feedback for the ‘cued+feedback' words differed between the two experimental groups. While the correct-feedback group (*N*=14) received the correct German translation after the Dutch word during sleep, the false-feedback group (*N*=13) received an incorrect German translation. Participants of the control group (*N*=16) received 40 Dutch cues, which were followed by late correct feedback (1,500 ms instead of 200 ms interstimulus interval) and 40 Dutch cues that were immediately followed by a pure sinus tone. In the cueing categories, the proportion of remembered and forgotten word pairs of the last learning trial was maintained. All words were exclusively replayed during NREM sleep. A cued recall procedure was applied after sleep testing the participant's memory for the German translations.

Twenty-seven healthy subjects participated in the two main experimental groups (correct-feedback group: *N*=14; false-feedback group: *N*=13). Another 16 participants took part in an additional control experiment. After the learning of 120 Dutch–German word pairs, subjects slept for 3 h in the sleep laboratory. We applied the so-called half-night paradigm, which has been repeatedly used successfully to demonstrate sleep-stage-specific effects on the consolidation of divers material^24^,^25^,^26^ to explicitly investigate the influence of NREM sleep on consolidation processes and to retain the identical experimental procedure as in our previous work^21^. During sleep stage N2 and slow wave sleep (SWS) a selection of the prior-learned vocabulary was replayed. In the correct-feedback group, Dutch cues replayed during sleep were immediately followed by the correct German translation. In the false-feedback group, Dutch cues were followed by an incorrect German translation. In both groups, one-third of the Dutch cues was always played without any feedback (cued words), and another third of the Dutch words was not played at all (uncued words). Before and after the sleep interval, memory for the German translations was tested, using a cued recall procedure (see Fig. 1, for a summary of the procedure).

### Effects of verbal cueing on memory for Dutch vocabulary

**Figure 2:**
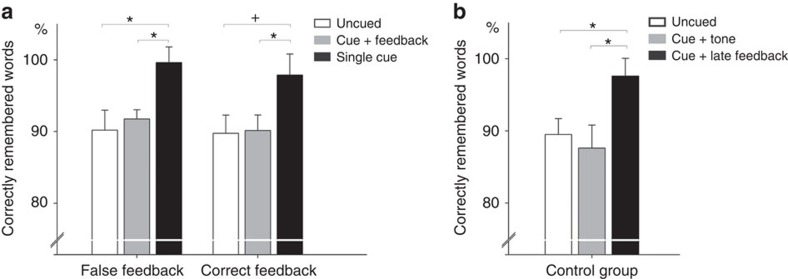
Behavioural results. (**a**) The same pattern of results is visible in both the correct (*N*=14) and the false-feedback group (*N*=13). A repeated measures ANOVA followed by *post hoc* comparisons revealed that in the false-feedback group memory for ‘cued' words (black bar) was enhanced as compared with ‘uncued' (white bar) and ‘cued +feedback' words (grey bar). In the correct-feedback group memory for ‘cued' words (black bar) was enhanced as compared with ‘cued +feedback' words (grey bar), while the difference to ‘uncued' words (white bar) reached a statistical trend (*P*=0.069; after correction for multiple comparisons). (**b**) For the control group (*N*=16) a very similar enhancing effect appeared when Dutch cues were followed by late and correct feedback (1,500 ms instead of 200 ms interstimulus interval) as compared with the ‘uncued' (white bar) and ‘cued+tone' words (grey bar). Retrieval performance is indicated as percentage of recalled German translations with performance before sleep set to 100%. Values are mean±s.e.m. *P≤0.05; ^+^*P*<0.07.

Consistent with our previous work^21^, re-exposure to Dutch cues during NREM sleep without additional feedback significantly improved memory recall after sleep in both the correct and the false-feedback group by roughly 10% points as compared with ‘uncued' words (98.78±1.78% versus 89.98±1.89%, *P*<0.001, see Fig. 2a). In contrast, providing auditory feedback after the Dutch cue completely blocked this beneficial effect of cueing during sleep: participants remembered only 90.98±1.22% word pairs of the ‘cued+feedback' category, which was significantly lower than memory for ‘cued' words without feedback (*P*<0.001). Performance level for ‘cued+feedback' words was basically identical as compared with uncued words (*P*=0.93, see Table 1 for descriptive values). The type of feedback was completely irrelevant for this effect: correct as well as false feedback after cueing during NREM sleep similarly blocked memory benefits both in the correct-feedback group (90.14±2.17%) as well as in the false-feedback group (91.75±1.29%, see Fig. 2b, for pairwise comparisons). Memory performance in the ‘cued+feedback' category did not differ between the experimental groups (*P*>0.50). The pattern of results was substantiated by a significant overall main effect of cueing category (‘cued+feedback', ‘cued' and ‘uncued', *F*_(2,50)_=13.34, *P*<0.001; *η*^2^=0.35) and no interaction with the factor ‘type of feedback' (correct versus false, *F*_(2,50)_=0.76, *P*>0.90).

Thus, our findings indicate that re-exposure to ‘cues+feedback' during sleep blocks the benefits of cueing, irrespective of whether the feedback is correct or false. The result pattern suggests that the mere presentation of a second auditory stimulus presented immediately after cueing disrupts underlying neural processes critical for memory benefits of cueing during NREM sleep. To further investigate this hypothesis we ran an additional control group (*N*=16) where correct feedback was delivered at a later point in time (1,500 ms instead of 200 ms after cue offset). Another third of the Dutch cues was directly followed by a tone to exclude any relevance of the verbal content of the feedback. In line with our main conclusion, re-exposure to Dutch cues followed by late feedback significantly improved memory recall after sleep as compared with ‘uncued' words (97.61±2.45% versus 89.51±2.21%, *P*=0.049, see Fig. 2b). Presenting a tone after the Dutch cue completely blocked this beneficial effect of cueing during sleep: participants remembered only 87.61±3.25% word pairs of the ‘cued+tone' category, which was significantly lower than memory for ‘cued+late feedback' words (*P*=0.027). Performance level for ‘cued+tone' words was highly similar to memory for uncued words (*P*=0.88, see Table 1 for descriptive values). Again a significant overall main effect of cueing category emerged (‘cued+late feedback', ‘cued+tone' and ‘uncued', *F*_(2,30)_=5.95, *P*=0.007; *η*^2^=0.28).

The results of the control group indicate that an interstimulus interval of 1,500 ms is sufficient to obtain reliable memory benefits by cueing during sleep. Still, there was no additive effect of replaying the whole memory representation as memory performance for the ‘cued+late feedback' did not differ from the ‘cued' condition of the preceding experimental groups (*P*=0.83). In contrast, presenting a tone directly after the Dutch cue again completely blocked the beneficial effects, reaching similar performance levels as obtained in the prior feedback groups (*P*=0.48). The results of the additional control group further strengthen our main conclusion that irrespective of its content, presenting a second auditory stimulus immediately after cueing interferes with neural processes critical for memory stabilization after cueing during NREM sleep (see Supplementary Table 1 and Supplementary Note 1, for sleep parameters and sleep-related analyses for all three experimental groups).

### Neural correlates of cueing during sleep

**Figure 3:**
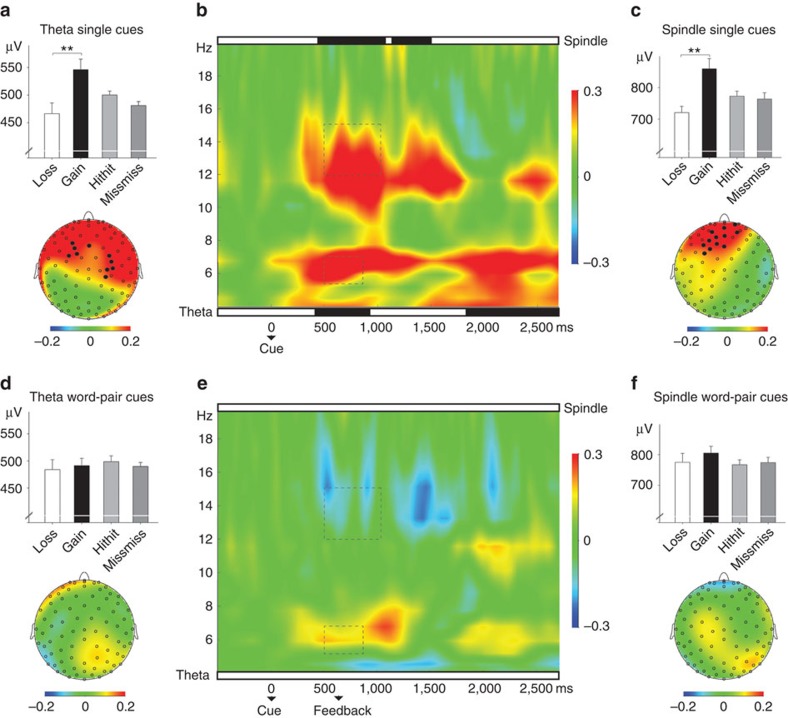
Results for oscillations in the theta and spindle range in both sleep groups (*N*=20). Oscillatory activity during vocabulary replay was specifically analysed with regards to words for which cueing during sleep resulted in a behavioural change in memory performance. Words, which were not remembered before sleep but correctly retrieved after sleep, were labelled as ‘Gains'. In contrast, ‘Losses' refers to words, which were successfully retrieved before sleep but not after the sleep interval. Words were indexed as ‘hithit' in case they were remembered before and after the sleep interval, while words neither retrieved before nor after sleep entered the category ‘missmiss'. Successful cueing of Dutch words was associated with enhanced power in the theta (**a**) and spindle (**c**) band. (**b**) Representative electrode F3. Verbal cues were presented at time 0 ms. The rectangle illustrates the time window used for the repeated measures ANOVA and *post hoc* comparisons in the bar chart. Top and bottom panels indicate significant differences in *post hoc t*-tests (in black) between ‘gains' and ‘losses' in spindle and theta power, respectively. (**d**–**f**) The differences in theta (**d**) and spindle band (**f**) vanished when cues were immediately followed by auditory feedback during sleep (that is, the false or correct German translation). Values are mean±s.e.m. ^**^P≤0.01.

To assess the blockade of cueing benefits on a neural basis, we analysed oscillatory responses to vocabulary cues during sleep (for analyses regarding event-related potentials see Supplementary Fig. 1 and Supplementary Note 2). Data of the correct- and false-feedback groups were collapsed, as the behavioural effects of cueing and feedback was identical in both groups. Based on our previously reported analysis and results^21^, we analysed oscillatory activity in the theta band in a time window of 500–800 ms after cue onset. Consistent with our previous findings, cued words without feedback that were correctly remembered after sleep were associated with an increased theta oscillation (∼6 Hz) as compared with the subsequently forgotten cued words (*P*=0.03). The effect was most pronounced when comparing cued words that were correctly recalled after but not before sleep (‘cued gains': 545.91±19.54 μV) with those that had been known before but not after sleep (‘cued losses': 466.46±19.44 μV, *P*=0.001; for details see Fig. 3). The categories ‘gains' and ‘losses' reflect a clear behavioural change after cueing, therefore best representing the neural pattern associated with processes underlying successful versus unsuccessful cueing for later memory retrieval (see Supplementary Table 2 and Supplementary Note 3, for a behavioural analysis of ‘gains' and ‘losses'). The increase in induced theta power between ‘cued gains' and ‘cued losses' had a stable fronto-central distribution (see Fig. 3). Furthermore, ‘cued gains' differed significantly in theta power from ‘cued hithit' (words remembered before and after sleep, *P*=0.016) and ‘cued missmiss' (words neither remembered before nor after sleep, *P*=0.002). To further investigate the exact time course of the effect we compared theta activity for ‘gains' and ‘losses' in 100 ms steps, ranging from 0 to 2,500 ms. The results indicated (after correcting for multiple comparisons), that theta activity associated with ‘gains' was significantly stronger as compared with ‘losses' in an early time window from 400 to 900 ms and in a late time window ranging from 1,800 to 2,500 ms (for details see Supplementary Table 3).

In contrast to ‘cued' words, no significant effect associated to theta power emerged for ‘cued+feedback' words presented during sleep. Theta activity between 500 and 800 ms after cue onset neither differed between subsequently remembered versus forgotten ‘cued+feedback' words (*P*=0.16) nor between ‘cued+feedback gains' versus cued+feedback losses' (*P*=0.74). Furthermore, no difference for ‘gains' and ‘losses' was observable when comparing theta activity in 100 ms steps after cue onset (all *P*>0.3). A direct comparison revealed that theta power associated with Dutch words without feedback (cued gains) was significantly stronger than theta power for Dutch words presented with feedback (‘cued+feedback gains', *P*=0.032; time window: 500–800 ms). This feedback-dependent difference in theta effects was further substantiated in an overall analysis of variance (ANOVA) by a significant interaction between the factors feedback (‘cued' versus ‘cued+feedback') and memory consequence (‘gains' versus ‘loss', *F*_(1,19)_=9.31, *P*=0.007; *η*^2^=0.31).

In addition, we explored possible oscillatory differences with regards to our conditions in the spindle frequency range (∼13 Hz). Similar to theta power, we observed a significant increase in spindle power in the time window 500–1,000 ms after cue onset for successfully remembered ‘cued' words as compared with later forgotten words (*P*=0.05). The effect was most pronounced for ‘cued gains' versus ‘cued losses' (*P*=0.005) and had a stable fronto-central distribution (see Fig. 3). ‘Cued gains' differed significantly from ‘cued hithit' (*P*=0.002) and ‘cued missmiss' (*P*=0.017). Again, we contrasted the temporal effects of ‘gains' and ‘losses' in 100 ms steps. ‘Gains' differed from ‘losses' in a broad time window from 400 to 1.500 ms after cue onset (for details see Supplementary Table 3).

Once more no significant effect associated to power in the spindle range emerged for ‘cued+feedback' words presented during sleep. Spindle power between 500 and 1,000 ms after cue onset neither differed between subsequently remembered versus forgotten ‘cued+feedback' words (*P*=0.23), nor between ‘cued+feedback gains' versus ‘cued+feedback losses' (*P*=0.16). Consequently, no difference between ‘gains' and ‘losses' was visible when comparing activity in the spindle range in 100 ms steps. Spindle power for gained ‘cued' words without feedback was higher as compared with gained ‘cued+feedback' words (*P*=0.05) and the interaction between the factors feedback (‘cued' versus ‘cued+feedback') and memory consequence (‘gains' versus ‘loss') was highly significant (*F*_(1,19)_=15.31, *P*=0.001; *η*^2^=0.32). Generally, oscillatory result patterns were highly similar in the additional control group with enhanced theta and spindle activity for cueing success in the ‘cued+late feedback' condition and no effects in case that the cue was followed by immediate feedback (see Supplementary Table 4 and Supplementary Note 4 and Supplementary Fig. 2). In an exploratory analysis, we investigated possible oscillatory differences with regards to our conditions in the high beta range (20–30 Hz). No differences emerged, indicating the specificity of effects in the theta and sleep spindle range. Finally, we counted frontal slow waves in a single-trial analysis and observed that ‘gains' were typically followed by an increased number of slow waves as compared with ‘losses' for both ‘cue' and ‘cue+feedback' conditions. However, the number of slow waves was highest for cued ‘gains' without feedback (see Table 2 and Supplementary Note 5).

If the presentation of feedback indeed blocked the neural correlates of successful memory cueing during sleep, it might be possible that the feedback itself acted as a memory cue. During learning, participants first heard the Dutch word and then recalled the German translation. In spite of this clear direction in the associative learning procedure, it might still be possible that the German translation also weakly reactivated the associated Dutch word during sleep.

**Figure 4:**
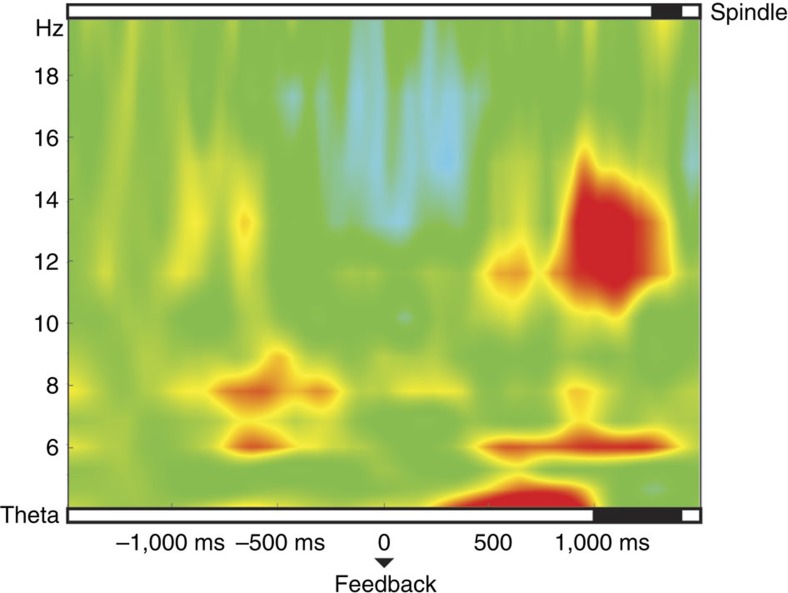
Oscillatory results for the recoded feedback. With regards to the feedback presented during sleep, theta and spindle power differed in a late time window (representative electrode Fz), when the categories of ‘gains' and ‘losses' were recoded in the false-feedback group to adequately reflect behavioural gains and losses with respect to the feedback (and not the first word, as in the original analysis). The zero point refers to the onset of the German feedback cue. Top and bottom panels indicate significant differences (in black) between ‘gains' and ‘losses'.

As false feedback was created by randomly intermixing Dutch–German pairs taken from original learning list, we additionally recoded the categories of ‘gains' and ‘losses' in the false-feedback group to adequately reflect behavioural gains and losses with respect to the feedback (and not the first word, as in the original analysis). ‘Gains' and ‘losses' of both experimental groups were again collapsed (for an analysis without recoding and a specific analysis for the correct-feedback group, see Supplementary Notes 6 and 7 and Supplementary Fig. 3). After recoding, the behavioural ‘gains' with respect to the feedback in both experimental groups were again associated with enhanced theta power in a late time window ranging from 1,000 to 1,400 ms (all *P<*0.03) after feedback onset. Spindle activity differed as well in a short, late time frame from 1,300 to 1,400 ms (*t*_19_=2.25; *P*=0.036; see Fig. 4). The re-occurrence of increased theta and spindle activity associated with behavioural ‘gains' after the feedback presentation again suggests that oscillatory processes might be important for successful memory cueing during sleep and should not be disturbed by further auditory input.

## Discussion

The present study contrasted for the first time the effects of presenting verbal cues versus cues followed by correct and false feedback during sleep on later memory performance. The replay of foreign vocabulary cues improved later memory performance. In sharp contrast, presenting auditory feedback (that is, German words) immediately after the Dutch cue completely blocked the beneficial effect of cueing during sleep on later recall, independent of whether the feedback (that is, the German translation) was correct or false. Furthermore, successful verbal cueing was associated with increased theta and spindle activity during NREM sleep. This neural pattern associated with cueing success vanished when verbal cues were directly followed by correct and false feedback, suggesting that auditory feedback after cueing disrupts neural and oscillatory processes critical for memory stabilization after reactivation during sleep.

An additional control group, where correct feedback was provided after a longer time delay, while other Dutch cues where directly followed by a tone, gave further insights into the temporal dynamics of crucial processes associated with reactivation processes during sleep. Here, the enhancing effect of cueing re-emerged in the case that feedback was provided after a delay of 1,500 ms, whereas the presentation of a tone directly after the Dutch cue as well blocked any behavioural effects. Again successful verbal replay was associated with increased theta and spindle activity during NREM sleep, whereas, this neural pattern completely vanished when verbal cues were followed by a tone.

The enhancing effect of replaying vocabulary cues during NREM sleep on later memory performance replicates our recent finding of memory improvements after cueing foreign vocabulary during sleep^21^. As in our former experiment, we explicitly chose Dutch as a foreign language to achieve a sufficiently high number of newly learned vocabulary required for our analysis after only two learning rounds before sleep. Whether the degree of prior knowledge of related languages influences the effectiveness of cueing during sleep is still an open question. In our study, the close relation between the languages Dutch and German might have enhanced cueing efficiency, since it is assumed that pre-existing schemata facilitate the integration of new memories into older neocortical traces^27^. Thus, replicating our results with more distant languages would be of importance with regards to this issue.

Still, our replication demonstrates the reliability of the beneficial effect of verbal cueing during sleep and adds further support to the assumptions of the active system consolidation theory, which postulates that spontaneous memory reactivations during sleep are essential for the beneficial effect of sleep on memory consolidation. Various studies have successfully used memory-associated cues such as odours or sounds to specifically strengthen declarative memories during sleep^17^,^18^,^28^. Here, we wanted to specifically test whether the beneficial effect of cueing during sleep would be altered if memory cues were followed by correct or false information. Thus, the original rationale of the study was based on ideas of the reconsolidation theory, assuming that reactivation (during wakefulness) renders a memory trace susceptible for updating depending on the incoming external feedback: if the incoming information does not match the reactivated memory traces, the reactivated and instable memory trace is assumed to be updated or possibly even forgotten. In case the feedback matches the reactivated memory trace, the representation is strengthened and reconsolidated^29^,^30^. Our pattern of results falsifies the predictions of the reconsolidation theory when cueing occurs during sleep, by showing that presentation of auditory information immediately after cueing completely blocks its beneficial effects, independent of the type of feedback.

Our behavioural results have at least two important implications: first, presentation of the whole memory content (that is, ‘cue+correct feedback') does not further strengthen memories during sleep, even when correct feedback is presented with a sufficient time delay. This result suggests that presentation of memory cues alone and the induction of unintentional memory reactivation appear to be necessary for the beneficial effects of cueing during sleep. Second, presentation of interfering information after cueing during sleep (cue+false feedback) does not further impair memory retention. While there are some reports showing that learning of new associations in simple conditioning paradigms might be possible during sleep^31^, our results provide no further support for the notion that presentation of interfering information after cueing induces forgetting due to newly learned and interfering associations.

Thus, the fact that correct and false feedback as well as a tone similarly blocked the beneficial cueing effect during sleep favours an alternative explanation: Based on our behavioural results pattern, it seems highly probable that the presentation of a second stimulus might have unspecifically blocked or disturbed neural processes elicited by the cue that are critical for the stabilization of reactivated memories during sleep. In contrast, when no feedback followed the cue, reactivation-associated processes were allowed to proceed uninterrupted, thereby exerting its beneficial effects on later memory performance. The finding that the beneficial effects of replay reappeared when correct feedback was presented at a later point in time (that is, 1,500 ms after cue offset) indicates that a certain sensitive plasticity period after the cue is necessary for stabilizing and strengthening the reactivated memory representation. The mere presentation of a second auditory stimulus, irrespective of its content, might have interfered with these plasticity processes, resulting in an absence of any behavioural memory improvement. Still, there was no behavioural difference between the ‘uncued' and ‘cue+feedback' conditions. This pattern of results might suggest that the feedback-related disruption only interfered with cue-related enhancement processes, but did not generally disrupt endogenous and spontaneous reactivation processes associated with the consolidation of certain words during sleep.

The interfering effect of the second auditory stimulus might have occurred on different levels: additional auditory input after cueing possibly disturbed ongoing reactivation processes, which are reported to persist until the next cue^32^ and also occur at a ca. 10 times faster time scale as external information processing^33^. Otherwise, unspecific auditory processing after cueing might have interfered with ongoing oscillations critical for stabilizing the reactivated memory. Our time frequency results appear to favour the second alternative. Successful cueing during sleep was accompanied by a post-stimulus increase in induced theta and spindle power. These effects were most pronounced when contrasting behavioural gains and losses. As those categories reflect a behavioural change after cueing, they might best represent the neural pattern associated with successful versus unsuccessful cueing for later memory retrieval. Furthermore, activity associated with successful cueings (gains) differed significantly from both other categories, namely ‘hithit' and ‘missmiss'. Still, it has to be noted that the neural correlates of ‘hithit' and ‘missmiss' words are more ambiguous. In these cases, cueing during sleep might have been ineffective for sufficiently strong memory traces (hithit) or non-existing associations (missmiss). Altogether, these results suggest that oscillations in the theta and sleep spindle range are strongly associated with the successful cueing of memories during sleep.

These effects almost completely vanished when a second stimulus was replayed directly after the Dutch cue. Cueing-associated neural processes were visible starting ∼500 ms after word onset. Our cueing procedure in the first two experimental groups was such that in case of cue+feedback, the feedback succeeded the cue at 200 ms. Depending on the length of the cue (varying between 450 and 700 ms), presentation time of feedback fell as a consequence into a time range between 650 and 900 ms. According to our cueing results, presentation of the feedback might have fallen into a critical time window, thereby blocking cueing-related stabilization processes associated with oscillations in the theta and spindle range. This interpretation is as well supported by the results obtained from our control group. Here correct German feedback was presented after a longer delay (∼1,500 ms after cue offset), which led again to enhanced activity in the theta and spindle range for gains as compared with losses. Furthermore, providing a tone directly after the cue as well blocked any oscillatory effects. Interestingly, slight increases in theta and spindle activity re-emerged after presentation of the feedback, suggesting that the German translation might have acted as memory cue to reactivate the associated Dutch word in a reversed order, although without any clear behavioural consequences. Future studies are required to address the important issue whether cueing during sleep is directional, that is, whether the memory benefits by cueing depend on the direction of the association learned during encoding before sleep.

It is assumed that the co-occurrence of memory reactivations with spindle-ripple events synchronized by slow oscillations is critical for stabilizing the reactivated memory trace^13^,^20^,^34^,^35^,^36^. Here, we did not synchronize the verbal cues to the phase of endogenous slow waves, however, successful reactivated memories were more often followed by slow waves. Nevertheless also losses—the behavioural category where cueing had clearly no beneficial effect—were followed by slow waves. Furthermore, the number of slow waves differed significantly between ‘gains' and ‘losses' also in the feedback conditions, which was not the case for theta and spindle activity (see Table 2). Accordingly, slow waves might represent a relevant pre-requisite for cueing success by providing the temporal frame for reactivation processes and the dialogue between the neocortex and subcortical structures, while activity in the theta and spindle range is critical for the stabilization/strengthening of reactivated memories.

The finding that successful cueing of Dutch words during sleep is related to post-stimulus increases in oscillatory sleep spindle power is also in line with their assumed involvement in processes of sleep-dependent memory consolidation^37^. Specifically, spindles have been associated with the learning of word pairs^22^,^23^, the integration of newly learned information into existing knowledge networks^38^ and reactivation processes in humans^36^, as well as in rodents^39^,^40^. Hence, numerous findings generally implicate sleep spindles in memory consolidation processes during sleep, which fits to our results and conclusions of a stabilizing role of oscillations in the spindle range on reactivated memory representations.

In addition, we found that successful cueing of Dutch words is accompanied by enhanced theta activity. As with spindle activity, successful cueing of Dutch words was associated with enhanced theta power, while no such difference was observable for cues followed by auditory feedback. The presentation of auditory feedback after the Dutch cue as well interrupted theta activity, which in turn blocked any behavioural effect of cueing during sleep.

The result that successful cueing during sleep is associated with enhanced theta power replicates recent findings in adults^21^. In addition, faster theta frequency or increased theta power during NREM sleep predicted better subsequent memory performance in patients with Alzheimer's disease or amnestic mild cognitive impairment and in healthy subjects^41^,^42^,^43^. A recent study demonstrated that spike timing during delta-nested theta rhythms controls a reciprocal interaction between deep and superficial cortical layers mimicking the alternating cortical dynamics of sensory and memory processing during wakefulness^44^. Despite the high relevance of theta oscillations in memory processing during wakefulness^45^,^46^,^47^ and growing evidence connecting theta activity during sleep and processes of memory reactivation/consolidation, theta oscillations have not yet been included in theoretical accounts of sleep and memory. Based on these recent findings, further examinations are needed to precisely determine the contribution of theta oscillations for stabilizing and strengthening memories during sleep.

While in our view the pattern of results fits best to the predictions of the active system consolidation theory^13^, our results might also be explained by alternative theoretical accounts. For example, the recent version of the synaptic homoeostasis theory assumes that reactivated memories during sleep are protected from synaptic downscaling during SWS (synaptic downselection)^48^. Thus, the reported memory benefits by cueing during sleep would then be a result of less-synaptic depression instead of strengthening of memory representations. Following this reasoning, providing auditory feedback after cueing disrupts this protective effect, probably by disrupting full activation of the memory representation necessary for the protection from synaptic downscaling. Alternatively, the opportunistic consolidation theory^49^ assumes that (NREM) sleep represents simply one of many possible time windows of improved memory consolidation due to reduced encoding of external information and reduced hippocampal plasticity. While we have shown that the reported cueing benefits are sleep specific^21^,^50^, according to this account they should similarly occur during periods of reduced encoding capacity induced, for example, by administration of benzodiazepines, alcohol or cholinergic antagonists. Future studies are needed to test this prediction. In contrast to these possible alternative explanations on the behavioural level, both theoretical accounts do not specifically predict our reported results on the oscillatory level. Particularly the increase in spindle activity associated with successful reactivation fits best with the assumptions of the active system consolidation theory.

In sum, our results demonstrate that the memory benefits of cued reactivation of foreign words during sleep and their oscillatory correlates are blocked when an additional auditory stimulus is presented immediately after the cue. Our results indicate that during a sensitive time period immediately after reactivation, certain plastic processes (probably associated with theta and spindle oscillations) are necessary to strengthen and stabilize the reactivated memory traces during sleep, leading to improved memory recall on the next day.

## Methods

### Subjects

Thirty healthy, right-handed subjects (19 females, mean age=22.00±2.6 years) with German mother tongue and without Dutch language skills participated in the study. Three subjects had to be excluded due to insufficient sleep. In the final sample, 14 volunteers participated in the correct-feedback group (10 females, mean age=22.7±3.09 years) and 13 subjects in the false-feedback group (9 females, mean age=21.15±1.95 years). Another 16 subjects took part in the control experiment (10 females, mean age 23.31±2.33 years). The three experimental groups were conducted sequentially. Age and gender distribution did not differ between the experimental groups (both *P*>0.75). The sample size of each experimental group was determined in accordance with previous human reactivation and sleep studies (for example, refs 17, 18, 21). These studies have revealed a large effect of reactivation during sleep on memory, which can be adequately detected in a sample of 12–17 participants in a repeated-measure design.

Subjects did not take any medication at the time of the experimental session and were free of any neurological or psychiatric disorders. All participants reported a good sleep quality. Furthermore, they had not been on a night shift for at least 8 weeks before the experiment. Subjects were briefed to wake up by 07:00 h and avoid caffeine on the morning of the experiment. The ethics committee of the Department of Psychology, University of Zurich approved the study. Before beginning the study, participants gave informed consent and received monetary compensation (120 Swiss francs) after completion.

### Design and procedure

All subjects arrived at the sleep laboratory at 21:00 h. The experimental session started with the set-up for polysomnographic recordings during which we applied electrodes for electroencephalographic (EEG), electromyographic (EMG) and electrocardiographic (ECG) recordings. Before the experiment, participants were habituated to the experimental setting by spending an adaptation night in the sleep laboratory. The temperature in the sleep lab was kept constant at 21 °C.

At around 22:00 h the experiment started with the learning of Dutch–German word pairs (for details see Vocabulary Learning Task). Following the learning phase, subjects entered the bed at ∼23:00 h and lights were turned off. All participants slept for ∼3 h. During NREM sleep (sleep stages N2 and SWS), a set of the Dutch words, which were learned before, was replayed for 90 min (cued words, see Reactivation of Vocabulary for details). Subjects were awoken at 2:00 h from light sleep (sleep stage N1 or N2) and after 15 min of recovery, cued recall performance for German translations of the Dutch words was assessed after sleep (see Cued Recall Testing after the Sleep Interval for details).

### Vocabulary learning task

Subjects had to learn 120 Dutch–German word pairs in the course of three learning rounds (the word pairs are specified in the Supplementary Table 5). All words were presented via loudspeaker (range of word durations: 450–700 ms; 70 dB sound pressure level). With regards to the first learning round, each trial consisted of a Dutch word, which was succeeded by a fixation cross for 200 ms. Afterwards, the German translation was presented via loudspeaker. Between trials a 1,000–3,000 ms intertrial interval was realized. The task of the participants was to commit as many word pairs as possible to memory.

The trials of second learning round started again with the presentation of the Dutch word. In this phase, Dutch words were followed by a question mark, with a time limit of 7 s. Within these 7 s, subjects had to vocalize the correct German translation (if possible) or to indicate that they were not able to do so by saying ‘next' (German translation: ‘weiter'). In any case, the correct German translation was presented afterwards.

The same cued recall procedure was again administered in the third learning round except that presentation of feedback of the correct German translation was omitted. The experimenter coded the correctness of each answer. Only answers, which completely matched the learned translations, were accepted as correct. The performance level obtained in the final learning round was used as baseline memory performance before the retention interval. On average participants correctly recalled 58.62±16.77 German translations out of the 120 word pairs (range 27–104 words). Thus, we achieved an optimal medium task difficulty (recall performance 48.85%), thereby excluding potential floor or ceiling effects. The baseline recall performance before sleep did not significantly differ between the three experimental groups (*F*_(2,40)_=1.22; *P*=0.35; see Table 1 for descriptive statistics).

### Reactivation of vocabulary

In the reactivation phase of the correct and false-feedback group the 120 Dutch–German word pairs were assigned to one of the three categories each consisting of 40 stimuli: ‘cued+feedback', ‘cued' and ‘uncued'. In the ‘cued+feedback' category, Dutch words were aurally presented repeatedly during NREM sleep directly followed by a German word. In the ‘cued' category, only Dutch words alone without any feedback were played, whereas uncued words were not presented during sleep. In all three categories, the proportion of remembered and forgotten word pairs of the last learning trial before sleep was maintained. Thus, all three categories comprised the same number of remembered and not remembered words before sleep. All words were individually and randomly chosen for each participant using an automatic MATLAB algorithm.

While the categories ‘cued' and ‘uncued' were identical for all participants, the type of feedback for the ‘cued+feedback' words differed between the correct- and the false-feedback group. The correct-feedback group received the correct German translation after the Dutch word during sleep. In contrast, the false-feedback group received an incorrect German translation. The false feedback was created by randomly intermixing the Dutch and German words of this category, thus new Dutch–German word combinations for replay during sleep were formed.

In total, 80 Dutch words (40 ‘cued+feedback' and 40 ‘cued' words) from the total of 120 Dutch–German word pairs were presented during NREM sleep via loudspeaker (50 dB sound pressure level). Each replay trial during sleep started with the presentation of a Dutch word. For ‘cued+feedback' trials, the Dutch word was followed after a 200-ms break by the respective German word. Thus each German word started 650–900 ms after the respective Dutch word onset. In the case of ‘cued' Dutch words, the second stimulus was replaced by a silent audio file (0 dB, duration: 600 ms).

With regards to the control group the same rationale of word selection for replay during the retention interval was administered. Thus, the 120 Dutch–German word pairs were assigned to one of the three categories each consisting of 40 stimuli: ‘cued+late feedback', ‘cued+tone' and ‘uncued'. The ‘cued+late feedback' category was basically identical to the one in the correct-feedback group, with Dutch words followed by their correct German translation. Importantly the interstimulus interval between the Dutch and the German word was lengthened from 200 to ∼1,500 ms after cue offset (2,000 ms apart from cue onset). In the ‘cued+tone' category Dutch cues were immediately followed by a sinus tone (length: 500 ms, 250 Hz), again with an inter-stimulus interval of 200 ms.

In all experimental groups, verbal cues presented during sleep were separated by an intertrial interval of 2,800–3,200 ms. Each word was replayed ∼17 times in a random order, resulting in 90 min of cued replay. We played words repeatedly during sleep in accordance with prior studies that successfully reactivated memories during sleep by presenting olfactory or auditory cues in a repeated fashion^17^,^21^,^28^,^51^. In addition, we wanted to make sure that we would obtain enough trials for the subsequent EEG analysis. Words were exclusively replayed during NREM sleep (sleep stages N2 and SWS), with sleep being permanently monitored by the attendant experimenter. The stimulation protocol was manually interrupted whenever signs of arousals, awakenings or REM sleep were visible. Word replay was stopped 5.8±0.4 times on average (see Supplementary Note 1 for further details).

### Cued recall testing after the sleep interval

During cued recall testing after sleep, Dutch words were again randomly presented via loudspeaker. After listening to the Dutch word, subjects were instructed to pronounce the correct German translation. Thus, the cued recall task after sleep was comparable to the final learning round before sleep. We calculated a relative retention score (that is, (post-sleep recall × 100)/pre-sleep recall) indexing recall performance of German translation, with memory performance before sleep set to 100%.

### Polysomnography

We used a high-density 128-channel Geodesic Sensor Net (Electrical Geodesics (EGI), Eugene, OR, USA) to record EEG. We kept impedances below 50 kΩ, which corresponds to the standards recommended by EGI. EEG signals were referenced to the vertex electrode (Cz) and sampled at a rate of 500 Hz. Furthermore, EMG and the ECG was recorded for standard polysomnography. In addition to our online sleep-stage identification, sleep architecture was determined offline according to the standard criteria^52^ by three independent raters. In a first step, two different raters scored sleep stages independently. They disagreed on ∼8% of the epochs. In cases of disagreement a third scorer made the final decision. Electrode sites F4, C4 and O2 referenced against average mastoids (electrodes 57 and 100) as well as HEOG (electrode site 1 referenced against electrode site 32) and VEOG (electrode site 25 referenced against electrode site 127) and EMG were used for offline sleep scoring. The three experimental groups did not differ in sleep architecture (see Supplementary Table 1).

### Oscillatory analysis

EEG signals were analysed offline using Brain Vision Analyzer 2.0 (Brain Products, Gilching, Germany). Initially, raw EEG data were re-referenced to the linked mastoids as well as low-pass filtered (30 Hz, roll-off 24 dB per octave) and high-pass filtered (0.1 Hz, roll-off 12 dB per octave). The continuous EEG was epoched into intervals from 1,000 ms before until 3,000 ms after cue onset. All data were baseline corrected using an interval from −1,000 to 0 ms. Trials with artefacts (for example, movement artefacts and so on.) were manually removed after visual inspection. Subsequently, all segments were categorized based on the subject's memory performance into words remembered after sleep and words not remembered after sleep. In addition, remembered words were further divided into words not remembered before sleep but remembered after the sleep interval (‘cued gains' and ‘cued+feedback gains', respectively) and words remembered before and after sleep (‘cued hithit', ‘cued+feedback hithit' words). Words, which were not remembered after the sleep interval, were divided into two categories of words successfully remembered before but not after sleep (‘cued losses' and ‘cued+feedback losses', respectively) and words neither remembered before nor after the sleep interval (‘cued missmiss', ‘cued+feedback missmiss' words). As a minimum number of trials in each category are required for a stable analysis (for example, *N*>8, see ref. 53), for the oscillatory analysis we had to exclude three subjects from the false feedback and four subjects from the correct-feedback group. Thus, the final sample for the oscillatory analyses consisted of *N*=20 participants (that is, 10 participants from each group).

For the control group epochs were as well categorized based on the performance between pre- and post-sleep tests. Later remembered words were separated in ‘cued+late feedback gains' and ‘cued+tone gains' and ‘cued+late feedback hithit', ‘cued+tone hithit' words. Later forgotten words were separated in ‘cued+late feedback losses', ‘cued+tone losses' and ‘cued+late feedback missmiss', ‘cued+tone missmiss' words. Five participants had to be excluded from the EEG analysis due to an insufficient number of trials, resulting in *N*=11 participants entering the EEG analysis.

We analysed oscillatory activity with regards to the theta (5–7 Hz) and spindle range (12–15 Hz). Following Klimesch^47^, we extracted rather narrow frequency bands for further analysis (theta: 5.81–6.03 Hz; spindle: 12.93–13.25 Hz), thereby reducing the danger that frequency-specific effects go undetected. We concentrated on theta activity as activity in this frequency band was strongly associated with cueing success when vocabulary was replayed during sleep in our previous study^21^. Sleep spindles were chosen due to their assumed crucial role with regards to memory consolidation processes during sleep. Oscillatory slow wave activity (0.5–4 Hz) was not analysed in the time–frequency analysis because the rather short interstimulus interval between word presentation during sleep allowed only a limited number of cycles resulting in border effects.

In the time–frequency analyses, we analysed the theta and spindle frequency range in 10 logarithmic steps using a continuous wavelet transformation (complex Morlet waveform, Morlet parameter *c*=7). We discarded 400 ms at the start and the end of each segment to prevent possible edge effects. Prior to the time–frequency analysis, evoked power was subtracted from each single trial to obtain a measure of induced power, which has been associated to the binding of distributed cortical representations^54^. We performed the wavelet analysis on single trials after each segment had been normalized with respect to the baseline window ranging from −300 to −100 ms. The single trials were averaged afterwards. Statistics were performed in two steps: to allow direct comparisons with our own results reported previously, we first restricted the statistical analysis to the specific time intervals used in our previous study (that is, 500–800 ms after cue onset for theta activity, 500–1,000 ms for spindle activity). In these analyses, we tested the difference between ‘gains' and ‘losses' using a false discovery rate of *P*<0.05. All electrodes that survived this threshold were averaged and compared also with ‘hithit' and ‘missmiss' conditions in an ANOVA approach. To illustrate the time–frequency results, we selected one representative electrode from the respective cluster. Second, to characterize the timing of potential effects more precisely, we compared activity for ‘gains' and ‘losses' in 100 ms steps. Again, we corrected for multiple comparisons using a false discovery rate of *P*<0.05. In an exploratory analysis, we performed the same procedure for the high beta range (complex Morlet waveform, frequency range from 20 to 30 Hz in 10 logarithmic steps, Morlet parameter *c*=7).

In addition, we were interested in potential effects of the feedback cues during sleep. Therefore we specifically analysed oscillatory activity with regards to the presentation of the German words in the theta and spindle range. German words entered the category ‘gains', when the correct pairing was not remembered before sleep but was remembered after sleep. Likewise German words entered the category ‘losses', when the correct pairing was remembered before sleep but not afterwards. Again potential effects were analysed in 100 ms steps.

For the control group, generally the same approach was used to compute oscillatory activity in the theta and spindle range. A broad fronto-central electrode cluster, derived from the results of preceding experimental groups, was defined as region of interest. As the centre frequency of theta was slightly lower as compared with the first experimental groups, a frequency range of 5.00–5.18 Hz was extracted for subsequent analysis. The range in the spindle band used for statistics was identical to the preceding analysis (12.93–13.25 Hz).

For methods concerning the analysis of event-related potentials in response to the vocabulary replay during sleep see Supplementary Methods.

### Slow wave analysis

EEG segments corresponding to the gain and loss trials (−1,000 to 3,000 ms) were band-pass filtered in the range of 0.5–4.0 Hz using a stopband of 0.1 and 10 Hz (Chebyshev Type II filter; MATLAB, The Math Works Inc, Natick, MA, USA). Afterwards, we visually identified slow waves at electrode sites Fz, F3 and F4. Slow waves were defined as waves exceeding −75 μV and a total time duration of over 500 ms. Furthermore, they had to initiate between 0 and 800 ms after stimulus onset.

### Statistical analysis

We analysed all data using ANOVAs for repeated measurements. In case of significant interactions, we further specified the interaction pattern by applying pairwise *post hoc t*-tests corrected for multiple comparisons (Sidak). An overall threshold of *P*=0.05 served as threshold for statistical significance.

## Additional information

**How to cite this article:** Schreiner, T. *et al*. Auditory feedback blocks memory benefits of cueing during sleep. *Nat. Commun.* 6:8729 doi: 10.1038/ncomms9729 (2015).

## Supplementary Material

Supplementary Information Supplementary Figures 1-3, Supplementary Tables 1-5, Supplementary Notes 1-7, Supplementary Methods, and Supplementary References.

## Figures and Tables

**Table 1:** Overview of memory performance.

	**Cue**	**Cue+feedback**	**Uncued**	***F***	***P*-Value**
*False-feedback group*					
Cued recall					
Learning	20.07±1.08	20.50±1.08	20.28±1.02	2.07	0.14
Retrieval	19.92±1.04	18.79±1.04	18.28±1.09	5.21	0.01*
Change	−0.15±0.40	−1.71±0.29	−2.00±0.61	7.91	0.002**
% Change	99.61±2.19	91.75±1.29	90.18±2.80	8.04	0.002**
					
*Correct-feedback group*					
Cued recall					
Learning	20.61±1.98	21.23±1.95	21.07±1.98	2.38	0.12
Retrieval	20.23±2.14	19.23±1.93	19.23±2.10	4.09	0.02*
Change	−0.38±0.54	−2.00±0.46	−1.84±0.44	7.44	0.003**
% Change	97.88±2.94	90.14±2.17	89.76±2.54	5.74	0.008**
	**Cue+late feedback**	**Cue+tone**	**Uncued**	***F***	***P*-Value**
*Control group*					
Cued recall					
Learning	18.12±1.32	17.75±1.29	17.81±1.29	3.61	0.04*
Retrieval	17.56±1.28	15.62±1.36	15.87±1.20	7.09	0.003**
Change	−0.68±0.48	−2.00±0.48	−1.87±0.37	3.37	0.04*
% Change	97.61±2.45	87.61±3.25	89.51±2.21	5.95	0.007**

Data are means±s.e.m.; numbers indicate absolute or relative values of correctly recalled words that were presented during the retention interval (one-third as cues, one-third as cues+feedback; 80 in total) or not (one-third uncued words; 40 in total). Please note that the false and the correct sleep group differed with regards to the feedback. While for the false sleep group incorrect feedback was replayed during sleep, feedback in the correct sleep group was correct. In the control group, word pairs were as well assigned to one of the three categories each consisting of 40 stimuli: one-third as cues+late feedback (1,500 ms instead of 200 ms intestimulus interval), one-third as cues+tone or not (one-third uncued words). For cued recall testing, number of correctly recalled words during the learning phase before and the retrieval phase after the retention interval are indicated. Change (% change) refers to the absolute (relative) difference in performance between learning and retrieval phases. *P<0.05; **P<0.01.

**Table 2:** Relative number of slow waves.

	**Cue**	**Cue from ‘cued+feedback'**	***t***	***P*-Value**	**Feedback from ‘cued+feedback'**	***t***	***P*-Value**
*Sleep group I+II*							
Gains	45.16±3.02	33.06±2.18	3.41	0.003**	39.84±2.16	2.41	0.049*
Losses	32.85±3.21	24.53±2.19	2.39	0.027*	27.84±81	1.39	0.183
	**Cue from ‘cued+late feedback'**	**Cue from ‘cued+tone'**	***t***	***P*-Value**	**Feedback from ‘cued+late feedback'**	***t***	***P*-Value**
*Control group*							
Gains	49.80±4.78	32.82±5.07	3.97	0.003^**^	39.44±3.92	2.25	0.048*
Losses	34.85±3.95	28.24±3.58	1.89	0.087	29.74±3.61	1.58	0.144

Data are means±s.e.m.; relative numbers of slow waves following word onset of cues (the percentage of word presentations, which were followed by slow waves) and separately for the cues and the feedback of the ‘cue+feedback' category. For sleep groups I+II (that is, false- and correct-feedback groups), first columns including *t*-and *P*-values correspond to differences in the appearance of slow waves between ‘cue' versus cue from ‘cued+feedback'. *t*- and *P*-values on the right side depict differences between ‘cue' and feedback from ‘cued and feedback'. For the control group, first columns including *t*-and *P*-values correspond to differences in the appearance of slow waves between cue from ‘cued+late feedback' versus cue from ‘cued+tone'. *t*- and *P*-values on the right side depict differences between cue ‘cued+late feedback' and Feedback from ‘cued and late feedback'. *P<0.05; **P<0.01.
