# Supplementary material for: Auditory feedback blocks memory benefits of cueing during sleep
Source: Nat Commun. 2015 Oct 28;6:8729. doi: 10.1038/ncomms9729 (PMC4640077; doi:10.1038/ncomms9729)
Supplement: Supplementary Information — Supplementary Figures 1-3, Supplementary Tables 1-5, Supplementary Notes 1-7, Supplementary Methods, and Supplementary References. [file ncomms9729-s1.pdf]

Supplementary Figure 1

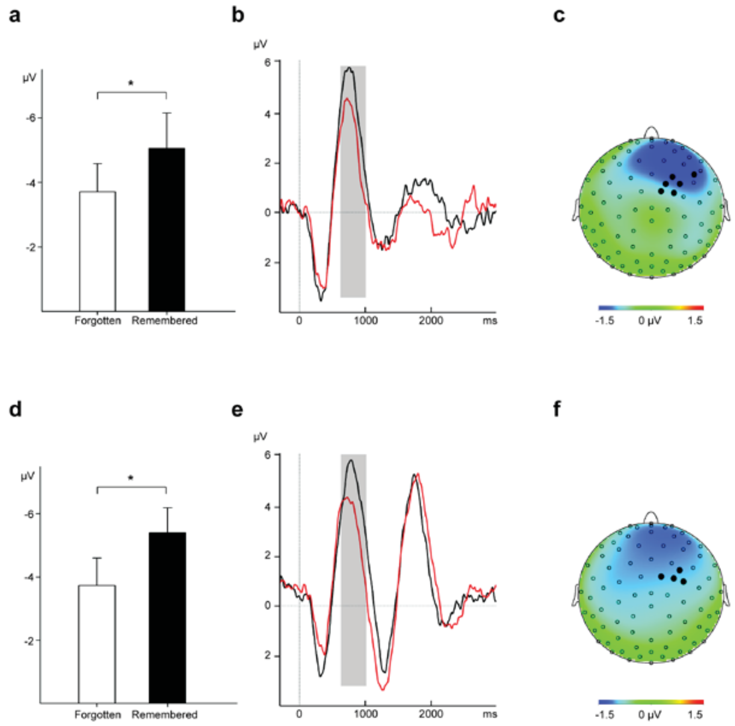

Results of event related potentials (ERPs) for cue presentation during sleep. **(a + b)** Later remembered and cued words were associated with a more pronounced negativity at frontal electrode sites (representative electrode E117). The rectangle illustrates the time window used for waveform quantification. **(c)** Scalp map representing the topographical distribution for the difference between "remembered" and "forgotten" in the time window between 700 and 1,000 ms, indicating a frontal distribution (all electrodes entered the analysis; black dots indicate significant electrodes at $P < 0.05$, false discovery rate) corrected for multiple comparisons). **(d - e)** The same pattern of results emerged for Dutch cues which where followed by feedback with a more pronounced negativity for later remembered as compared to forgotten words. Values are mean ± s.e.m. *: $P < 0.05$.

Supplementary Figure 2

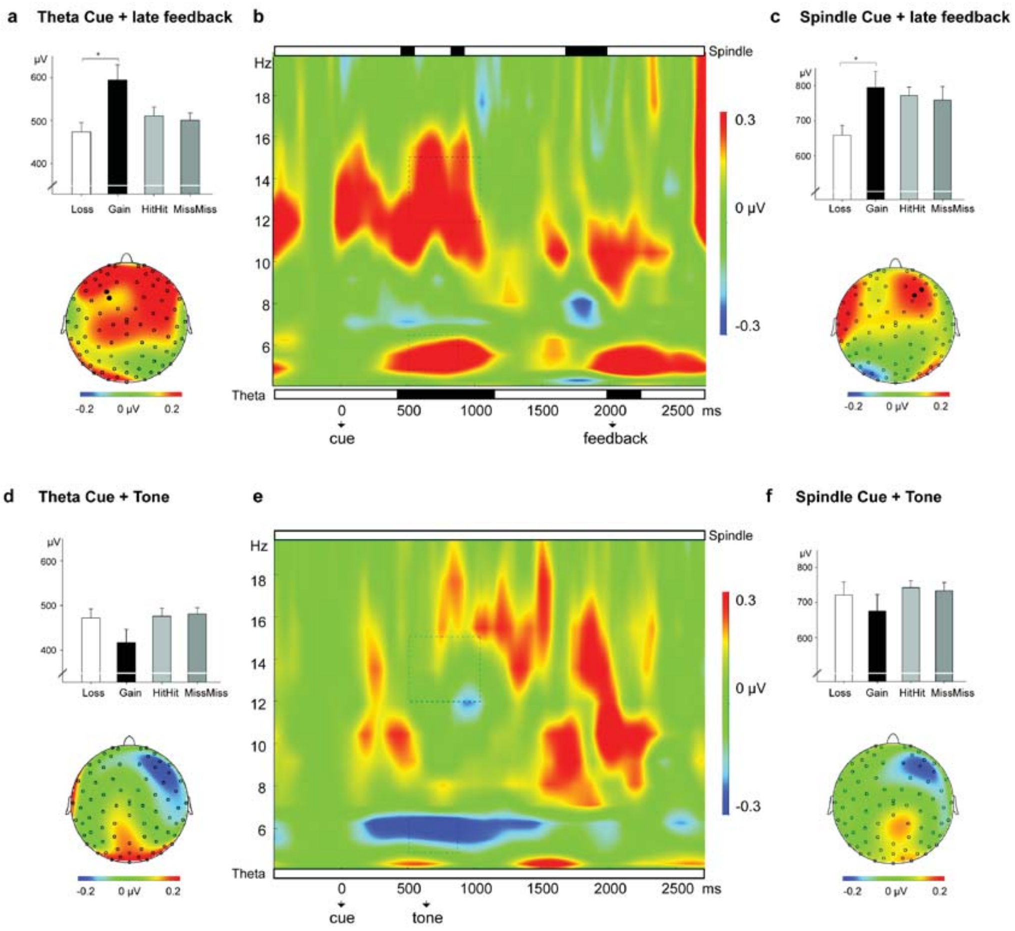

Oscillatory results for the control group. Oscillatory theta and spindle power, recorded during verbal replay in the control group (N=11), were computed for words, for which cueing during sleep led to a change in memory performance. "Gains" reflect cued words not remembered in the pre-sleep test but correctly recalled in the post-sleep test. "Losses" refer to cued words remembered in the pre-sleep test but not in the post-sleep test. Words remembered before and after the retention interval were labeled "hithit" and words not remembered both before and after the retention interval were labeled "missmiss". Successful replay of Dutch words followed by late correct feedback was associated with enhanced power in the theta **(a)** and spindle **(c)** band. **(b)** Representative electrode F3. Dutch cues were presented at time 0 ms, while German feedback was presented 2,000ms afterwards (i.e., 1,500ms after stimulus offset). The rectangle illustrates the time window used in the bar chart. Top and bottom panels indicate significant differences (in black) between "gains" and "losses" in spindle and theta power,

respectively. **(d - f)** The differences in theta **(d)** and spindle band **(f)** vanished when cues + tones (inter-stimulus-interval: 200ms) were replayed during sleep. Values are mean ± s.e.m. *: $P < 0.05$.

Supplementary Figure 3

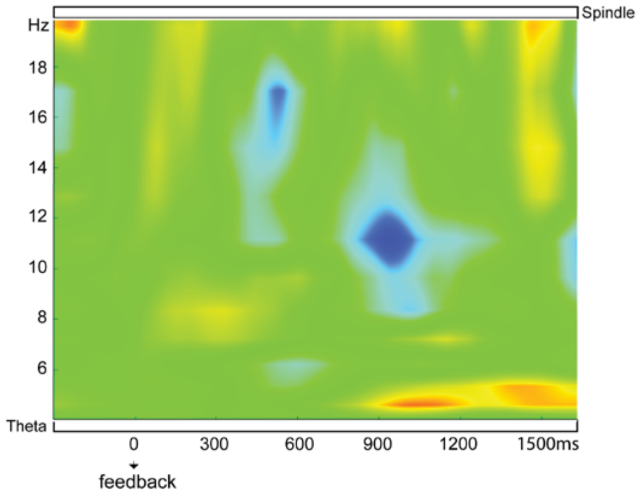

Oscillatory results for feedback presentation without recoding. With regards to the feedback presented during sleep, theta and spindle power did not differ (representative electrode F3). The zero point refers to the onset of the German feedback cue. Please note that in contrast to the oscillatory feedback results presented in the main text, here no behavioural recoding took place.

Supplementary Table 1. Sleep parameters for all three experimental groups

	False feedback group	Correct feedback group	Control group	*P*
Duration [min]				
N 1	9.21 ± 1.74	11.46 ± 2.36	11.62 ± 1.87	0.63
N 2	97.42 ± 4.81	102.03 ± 3.85	87.34 ± 6.41	0.14
SWS	62.78 ± 4.18	58.34 ± 3.46	66.84 ± 5.1	0.39
REM	20.85 ± 2.76	24.69 ± 2.73	21.56 ± 2.39	0.47
WASO	1.35 ± 0.47	2.37 ± 1.05	5.15 ± 1.84	0.29
Total	192.84 ± 4.51	200.34 ± 4.55	193.46 ± 4.8	0.48
Duration [%]				
N 1	4.65 ± 0.77	5.51 ± 1.01	5.91 ± 0.87	0.59
N 2	50.70 ± 1.99	51.01 ± 1.84	45.10 ± 3.07	0.18
SWS	32.97 ± 2.29	29.76 ± 1.81	34.17 ± 2.68	0.26
REM	10.41 ± 1.42	12.29 ± 1.09	11.17 ± 1.2	0.59
WASO	2.48 ± 0.68	2.26 ± 1.05	2.49 ± 0.86	0.31

Data are means ± s.e.m. N1, N2: NREM sleep stages N1 & N2, SWS: slow-wave sleep (N3), REM: rapid eye movement sleep, WASO: wake after sleep onset.

Supplementary Table 2: Behavioral data of “gains” and “losses” for cues, cues + feedback and uncued words in both experimental groups.

		Cued	Cued + feedback	Uncued	*F*	*P*
Gains						
	Sleep group I	2.35 ± 0.22	1.57 ± 0.34	1.42 ± 0.25	3.83	0.03*
	Sleep group II	2.00 ± 0.11	1.38 ± 0.18	1.46 ± 0.21	4.17	0.02*
	Control group	2.31 ± 0.41	1.37 ± 0.31	1.81 ± 0.35	2.21	0.12
Losses						
	Sleep group I	2.50 ± 0.31	3.28 ± 0.47	3.42 ± 0.50	2.83	0.07
	Sleep group II	2.38 ± 0.50	3.38 ± 0.48	3.30 ± 0.44	2.36	0.10
	Control group	2.81 ± 0.27	3.50 ± 0.45	3.37 ± 0.46	0.91	0.41
Gains minus Losses						
	Sleep group I	-0.15 ± 0.40	-1.71 ± 0.28	-2.00 ± 0.61	7.91	0.002**
	Sleep group II	-0.38 ± 0.54	-2.00 ± 0.46	-1.84 ± 0.46	7.44	0.003**
	Control group	-0.68 ± 0.48	-2.00 ± 0.48	-1.87 ± 0.37	3.37	0.047*

Data are means ± s.e.m; Sleep group I: False feedback group. Sleep Group II: Correct Feedback group. “Gains”: cued words, which were not remembered in the pre- but remembered in the post-sleep test. “Losses”: cued words, which were remembered before sleep, but not after sleep. *: $P < 0.05$; **: $P < 0.01$.

Supplementary Table 3: Significant time-windows comparing "gains" and "losses" for theta and spindle range for cues without feedback in the collapsed false and correct feedback groups

Gain / loss theta	time window	*t*	*P*
	400-500ms	3.73	0.001*
	500-600ms	4.34	< 0.001*
	600-700ms	4.22	< 0.001*
	700-800ms	3.44	0.003*
	800-900ms	2.77	0.01*
	900-1000ms	2.37	0.028
	1000-1100ms	2.33	0.031
	1100-1200ms	2.66	0.015
	1200-1300ms	2.98	0.023
	1300-1400ms	2.51	0.021
	1400-1500ms	1.99	0.060
	1500-1600ms	1.93	0.68
	1600-1700ms	2.09	0.050
	1700-1800ms	2.35	0.030
	1800-1900ms	2.85	0.01*
	1900-2000ms	3.36	0.003*
	2000-2100ms	3.34	0.003*
	2100-2200ms	3.24	0.004*
	2200-2300ms	3.09	0.006*
	2300-2400ms	2.98	0.008*
	2400-2500ms	2.55	0.01*

Gain / loss spindle	time window	*t*	*P*
	400-500ms	2.85	0.01*
	500-600ms	3.35	0.003*
	600-700ms	4.04	0.001*
	700-800ms	2.89	0.009*
	800-900ms	2.82	0.01*
	900-1000ms	3.15	0.005*
	1000-1100ms	2.91	0.009*
	1100-1200ms	2.21	0.04
	1200-1300ms	2.91	0.009*
	1300-1400ms	2.76	0.01*
	1400-1500ms	2.46	0.01*
	1500-1600ms	2.50	0.02
	1600-1700ms	2.16	0.04
	1700-1800ms	0.58	0.56
	1800-1900ms	-0.31	0.72
	1900-2000ms	-0.26	0.79
	2000-2100ms	0.91	0.37
	2100-2200ms	0.37	0.57
	2200-2300ms	0.29	0.77
	2300-2400ms	0.60	0.55
	2400-2500ms	-1.61	0.12

* indicate significant *P*-values surviving FDR correction. No significant time segments emerged in the "cue + feedback" condition (not shown).

Supplementary Table 4: Significant time-windows comparing "gains" and "losses" for theta and spindle range for the control group in the cue with late feedback condition.

**Gain / loss theta**	**time window**	***t***	***P***
	400-500ms	1.96	0.036
	500-600ms	2.67	0.016
	600-700ms	3.13	0.005
	700-800ms	3.50	0.003*
	800-900ms	2.75	0.01*
	900-1000ms	2.18	0.025
	1000-1100ms	1.88	0.044
	1100-1200ms	1.44	0.089
	1200-1300ms	0.52	0.304
	1300-1400ms	0.39	0.352
	1400-1500ms	0.71	0.246
	1500-1600ms	1.29	0.112
	1600-1700ms	1.38	0.098
	1700-1800ms	0.98	0.174
	1800-1900ms	1.15	0.137
	1900-2000ms	1.66	0.063
	2000-2100ms	1.98	0.042
	2100-2200ms	1.84	0.047
	2200-2300ms	1.55	0.075
	2300-2400ms	1.13	0.141
	2400-2500ms	0.94	0.145

**Gain / loss spindle**	**time window**	***t***	***P***
	400-500ms	2.26	0.023
	500-600ms	1.67	0.062
	600-700ms	1.21	0.127
	700-800ms	1.24	0.120
	800-900ms	2.07	0.032
	900-1000ms	0.86	0.203
	1000-1100ms	0.99	0.171
	1100-1200ms	1.55	0.075
	1200-1300ms	0.75	0.233
	1300-1400ms	0.67	0.258
	1400-1500ms	0.52	0.370
	1500-1600ms	0.20	0.421
	1600-1700ms	0.70	0.247
	1700-1800ms	2.91	0.0059*
	1800-1900ms	3.13	0.0052*
	1900-2000ms	2.03	0.035
	2000-2100ms	-1.17	0.133
	2100-2200ms	-0.91	0.190
	2200-2300ms	-0.46	0.325
	2300-2400ms	-0.37	0.298
	2400-2500ms	-0.57	0.322

* indicate significant *P*-values surviving FDR correction. No significant time segments emerged in the "cue + condition" condition (not shown).

Supplementary Table 5: Dutch words and German translations used in the learning task (English translations in brackets).

aarde	Erde (ground)	hoek	Ecke (corner)	rubber	Gummi (rubber)
amandel	Mandel (almond)	hout	Holz (wood)	schouder	Schulter (shoulder)
aroma	Geruch (odour)	huis	Haus (house)	schrift	Heft (booklet)
baars	Barsch (harsh)	ijskast	Kuehlschrank (fridge)	schuur	Schuppen (shed)
bagage	Gepaeck (lugagge)	ijzer	Eisen (iron)	servet	Serviette (napkin)
been	Knochen (bone)	inkt	Tinte (ink)	slot	Schloss (lock)
berk	Birke (birch)	kerk	Kirche (church)	sluis	Schleuse (sluice)
beugel	Buegel (bail)	kermis	Jahrmarkt (fair)	snavel	Schnabel (beak)
bezem	Besen (broom)	ketting	Kette (chain)	snuit	Schnauze (snout)
bijl	Axt (axe)	kogel	Kugel (ball)	soep	Suppe (soup)
bloes	Bluse (blouse)	koor	Chor (choir)	speeksel	Speichel (saliva)
boon	Bohne (bean)	kraag	Kragen (collar)	spijs	Brei (puree)
boord	Ufer (border)	kraal	Koralle (coral)	steen	Stein (stone)
borst	Brust (chest)	krijt	Kreide (chalk)	steiger	Steg (gangplank)
boter	Butter (butter)	kuit	Laich (spawn)	stof	Staub (dust)
burcht	Burg (castle)	kus	Kuss (kiss)	stoom	Dampf (stoom)
dak	Dach (roof)	landschap	Landschaft (landscape)	straat	Strasse (street)
dal	Tief (trough)	lat	Latte (lath)	strook	Streifen (stripe)
deur	Tür (door)	lepel	Loeffel (spoon)	tafel	Tisch (table)
dijk	Teich (pond)	lever	Leber (liver)	tegel	Fliese (slab)
doek	Tuch (cloth)	luis	Laus (louse)	trui	Trikot (jersey)
dorp	Dorf (village)	maag	Magen (stomach)	twijg	Zweig (twig)
draad	Faden (twine)	maaltijd	Essen (food)	vaas	Vase (vase)
draak	Drache (dragon)	mees	Meise (chicadee)	vakantie	Ferien (vacation)
droom	Traum (dream)	meeuw	Moewe (gull)	veer	Feder (feather)
duim	Daumen (thumb)	melk	Milch (milk)	vijg	Feige (fig)
eiwit	Eiweiss (protein)	moeras	Sumpf (swamp)	vorst	Frost (cold)
fles	Flasche (bottle)	molen	Muehle (mill)	vuur	Feuer (fire)
folder	Prospekt (brochure)	munitie	Munition (munition)	walm	Qualm (fume)
gat	Loch (hole)	munt	Muenze (coin)	werf	Hof (yard)
gijzelaar	Geisel (hostage)	naam	Name (name)	werktuig	Werkzeug (tool)
gist	Hefe (yeast)	navel	Nabel (navel)	wil	Rad (wheel)
goud	Gold (gold)	olijf	Olive (olive)	wol	Wolle (wool)
graat	Graete (fishbone)	onderdak	Unterkunft (housing)	woordenboek	Wörterbuch (dictionary)
graf	Grab (grave)	paart	Pferd (horse)	worst	Wurst (sausage)
griep	Grippe (flu)	peer	Birne (pear)	zak	Tasche (bag)
grond	Boden (floor)	penseel	Pinsel (brush)	zitkamer	Wohnzimmer (lounge)
hak	Haken (hook)	pols	Puls (pulse)	zeep	Seife (soap)
hei	Heide (heather)	rasp	Reibe (grater)	zoogdier	Saeugetier (mammal)
heuvel	Huegel (hill)	rits	Reihe (row)	zwaan	Schwan (swan)

Supplementary Note 1. Sleep and cueing

Online monitoring as well as offline analysis of the sleep EEG assured that presentation of auditory cues occurred during NREM sleep and did not lead to increased awakening responses (see Supplementary Table 1, for sleep data). Sleep architecture did not significantly differ between the three experimental groups (all $P > 0.18$). We excluded the occurrence of brief awakenings by showing that occipital alpha power 1000ms before and after each auditory cue did not differ (mean alpha power at electrode Oz: 1.37 ± 0.38 µV vs. 1.34 ± 0.28 µV; $t_{26} = 0.01$, $P = 0.92$).

To investigate whether the success of verbal cues was related to time spent in a certain sleep stage, we computed a memory advantage score (i.e., by subtracting memory for cued minus uncued words [1]) and correlated this score with the relative time spent in each sleep stage for all participants. We did not observe any significant associations (N2: r = 0.006, P = 0.96; SWS: r = 0.05, P = 0.51; REM: r = -0.11, P = 0.48).

Supplementary Note 2. ERP's associated with cueing during sleep

First, we analyzed ERPs for later remembered as compared to later forgotten cued words without feedback. In addition, we separated later remembered words in "cued gains" (i.e., cued Dutch words not remembered before sleep but correctly recalled after sleep) and "cued hithit" words (i.e., cued Dutch words remembered before and after sleep). Later forgotten words were separated in "cued losses" (i.e., cued words correctly retrieved before sleep but not remembered after sleep) and "cued missmiss" words (i.e., cued Dutch words not remembered before and after sleep). Consistent with our previous work[2] cued words without feedback that were correctly remembered after sleep were associated with an increased late negativity (700 – 1.000ms) in ERP's as compared to subsequently forgotten cued words ($F_{1,18}$ = 5.08; P = 0.03; for illustration please see Supplementary Fig. 1). Still, the effects of cueing on event related potentials was not as stable and reliable as in our recent study, as neither the comparison of "cued gains" and "cued losses", nor of "cued hithit" and "cued missmiss" words reached significance (both $P > 0.1$).

As for the “cued” words, ERP’s associated with “cued + feedback” words presented during sleep differed between subsequently remembered vs. forgotten “cued + feedback” words ($F_{1,18}$ = 7.76; P = 0.012), while no effect emerged between “cued + feedback gains” vs. cued + feedback losses” (P = 0.74) and “cued + feedback hithit” and “cued + feedback missmiss” (both P > 0.1). In order to examine whether the presentation of correct and false feedback was associated with differing ERP effects, we additionally analyzed ERP’s associated with the presentation of feedback. As with the preceding analysis we used a same time window ranging from 700 – 1000ms after feedback onset and applied the factor “group” as between subjects factor to investigate potential effects of the type of feedback on the ERP amplitude. None of the comparisons reached significance (all P > 0.2). Additionally, inspired by work concerning the neural correlates of prediction errors during wake, we analyzed a time window ranging from 200 to 300ms after feedback onset, corresponding to the feedback related negativity[3,4]. Again, we could not find any significant differences between conditions (all P > 0.5).

In sum, ERP’s for later remembered as compared to later forgotten were associated with a more pronounced late negativity, irrespective whether the Dutch cue was followed by feedback or not. Furthermore, “gains” and “losses”, as well as “hithit” and “missmiss” did not significantly differ in both conditions (“cued” and “cued + feedback”) and generally no difference emerged for the ERP’s concerning the feedback analysis. Thus, the late negativity obtained after cueing during sleep appears to be related rather to subsequently successful memory retrieval after sleep and does not capture processes of successful reactivation and stabilization by cueing. These results suggest that oscillatory activity associated with the cueing of memories during sleep is a more sensitive measure of the neural correlates of cueing success. In contrast to the ERP’s, activity in the theta and spindle range mirrored the beneficial effects of cueing and the blockade of memory benefits when delivering feedback in a very fine-grained and stable manner. Thus for future memory cueing studies, the analysis of oscillatory activity appears to provide more reliable means for picturing associated neural patterns.

Supplementary Note 3. Behavioral analysis of "gains" and "losses"

On the behavioral level, we analyzed "gains" and "losses" in both experimental sleep groups I and II (*false feedback and correct feedback groups, respectively).* The results revealed that cueing Dutch words during sleep increased the number of "gains" and marginally reduced the number of "losses" in both sleep groups (2-way interaction between the factors "gains vs. losses" and "cueing procedure"; $F_{(2,52)}$ = 15.53, P < 0.001). Additional analysis indicated that in both sleep groups, "Gains" differed significantly between the categories "cued", "cued + feedback" and "uncued" (both P < 0.02, see Supplementary Table 2), with significantly more "gains" in the cue condition, while "losses" reached a trend in the reverse direction (*sleep group I*: P= 0.07; *sleep group II*: P = 0.1). The same pattern of results emerged with regards to the control group, with a significant 2-way interaction between the factors "gains vs. losses" and "cueing procedure" ($F_{(2,30)}$ = 3.45, P = 0.045). Still, neither "gains" differed significantly between the categories "cued", "cued + feedback" and "uncued" (P = 0.12), nor "losses" (P = 0.41).

Supplementary Note 4. Oscillatory results of the control group

In the additional control group, we also analyzed oscillatory activity in the theta band in a time window of 500ms – 800ms after cue onset. Consistent with our results obtained from the previous single cue conditions, theta activity was enhanced for "gains" ("cued + late feedback gains" vs. "cued + late feedback losses": P = 0.04), with a similar fronto - central distribution (see Supplementary Fig. 2). When comparing theta activity for "gains" and "losses" in 100ms steps (ranging from 0 to 2,500ms) theta activity associated with "gains" differed from "losses" in an early time-window from 400ms to 1,000ms and in a late time window ranging from 2,000 - 2,200ms (for details see Supplementary Table 4).

In contrast, no significant effect associated to theta power emerged for "cued + tone" words presented during sleep, which is in line with the previous "cued + feedback" conditions. Theta activity between 500-800ms after cue did not differ between "cued + tone gains" vs. cued + tone losses" (P = 0.61). Additionally no difference for "gains" and "losses"

was observable when comparing theta activity in 100ms steps after cue onset (all $P > 0.2$). The feedback-dependent difference in theta-effects, reflecting the results from the behavioral analysis, was again confirmed in an overall ANOVA by a significant interaction between the factors feedback ("cued + late feedback" vs. "cued + tone") and memory consequence ("gains" vs. "loss", $F_{(1,10)} = 13.55$, $P = 0.004$; $\eta^2 = 0.57$).

Similarly, we again observed a significant increase in spindle power in the time window 500 – 1,000ms after cue onset for "cued + late feedback gains" vs. "cued + late feedback losses" ($P = 0.013$). When using 100ms steps, "gains" differed from "losses" between 400 and 500ms, 800 and 900ms and in a late time window from 1,700 to 2,000ms after cue onset (for details see Supplementary Table 4). In contrast for "cued + tone" words, spindle power between 500-1,000ms after cue onset did not differ between "cued + tone gains" vs. cued + tone losses" ($P = 0.1$), and no difference between "gains" and "losses" was visible when comparing activity in the spindle range in 100ms steps. Spindle power for gained "cued" words without feedback was higher as compared to gained "cued + feedback" words ($P = 0.03$) and the interaction between the factors feedback ("cued + late feedback" vs. "cued + tone") and memory consequence ("gains" vs. "loss") was highly significant ($F_{(1,10)} = 10.01$, $P = 0.01$; $\eta^2 = 0.5$).

Supplementary Note 5. Cueing and slow waves

In a single trial analysis, we counted the number of clearly identifiable slow waves (0.5 – 4 Hz) that followed cueing of "cued gains" words as compared to "cued losses", as well as the slow waves following the cues and the feedback, respectively of our "cued + feedback gains" as compared to "cued + feedback losses" categories. "Gains" were generally followed by an increased number of slow waves ($F_{(1,19)} = 36.33$, $P < 0.001$, main factor "gain/loss"), while "cued gains" were associated with the highest number of slow waves, following the word presentation ($F_{(2,38)} = 9.56$, $P > 0.001$, main factor "condition"). Cues followed by feedback presentations were associated with the smallest number of slow waves (cued gains from "cued + feedback" vs. "cued gains": $t_{19} = 3.41$; $P = 0.003$; "cued gains" vs.

"feedback gains" with regards to "cued + feedback": t_{19} = 2.41; P = 0.049), indicating that the presentation of feedback might have partly suppressed the emergence of slow waves.

A highly similar pattern of results was observable for the control group, with "gains" being followed by an increased number of slow waves ($F_{(1,10)}$ = 6.59, P = 0.028, main factor "gain/loss"), while "cued + late feedback gains" were associated with the highest number of slow waves, following the word presentation ($F_{(1,10)}$ = 20.67, P = 0.001, main factor "condition"). Cues followed by a tone were associated with the smallest number of slow waves (cued gains from "cued + late feedback" vs. "cued + tone gains": t_{10} = 3.97; P = 0.003; "cued + tone gains" vs. "feedback gains" with regards to "cued + late feedback": t_{10} = 2.25; P = 0.048; for descriptive data see Table 2).

Supplementary Note 6. Oscillatory activity after feedback during sleep

We investigated whether theta and spindle activity would generally differ between gains and losses after feedback presentation. We used the same time windows as in our main analysis (theta: 500 – 800ms after feedback onset; spindle: 500 – 1,000ms after feedback onset and a subsequent analysis in 100ms steps). We could not find any differences for theta and spindle activity after feedback onset with regards to gains and losses (all $P > 0.3$; for illustration see Supplementary Fig. 3). Thus, oscillatory activity after feedback presentation, irrespective whether the feedback was correct or false, did not reliably differentiate between our conditions.

Supplementary Note 7. Oscillatory activity after correct feedback during sleep

In an additional analysis we investigated whether theta and spindle activity would specifically differ between conditions following the presentation of correct feedback. We used the same time windows as in our main analysis (theta: 500 – 800ms after feedback onset; spindle: 500 – 1,000ms after feedback onset; for both theta and spindle activity a more detailed analysis in 100ms steps). We could not find any differences for theta and spindle activity after feedback onset with regards to gains and losses (all $P > 0.3$). We could not find any stable differences

neither in theta activity (time range 500 – 800ms; $t_9 = 0.23$; $P = 0.82$; all 100ms step comparisons $P > 0.17$), nor in spindle activity (time range 500 – 1000ms; $t_9 = -0.83$; $P = 0.42$; all 100ms step comparisons $P > 0.29$). Still, these results have to be interpreted with caution, as they were only based on 11 participants (in contrast to 20 subjects in all other analyses).

Supplementary Methods:

Event related potentials

Off-line EEG analysis was realized using Brain Vision Analyzer software (version: 2.0; Brain Products, Gilching, Germany). Data were re-referenced to averaged mastoids, low-pass filtered with a cut-off frequency of 30 Hz (roll-off 24 dB per octave) and high-pass filtered with a cut-off frequency of 0.1 Hz (roll-off 12 dB per octave). The EEG data was epoched into 3.3000ms segments beginning 300ms before cue onset. The 300ms interval preceding stimulus onset served as baseline and was used for baseline correction. All segments were visually inspected and major artifacts (e.g., due to movement) were rejected manually. Identical to the oscillatory analysis in the main text epochs were categorized based on performance between pre- and post-sleep tests yielding the following categories: We separated later remembered words in “cued gains” and “cued + feedback gains” (i.e., cued words and cued + feedback not remembered before sleep but correctly recalled after sleep) and “cued hithit”, “cued + feedback hithit” words (i.e., cued Dutch words and cued + feedback remembered before and after sleep). Later forgotten words were separated in “cued losses” and “cued + feedback losses” (i.e., cued words and cued + feedback correctly retrieved before sleep but not remembered after sleep) and “cued missmiss”, “cued + feedback missmiss” words (i.e., cued Dutch words and cued + feedback not remembered before and after sleep). Signal averaging was carried out separately per subject and per condition and grand averages of all conditions were calculated. For statistical analysis average EEG amplitudes measured over the interval from 700 to 1.000ms after stimulus onset were compared. To protect against error inflation due to multiple testing of multiple

electrodes, we used a false discovery rate (FDR) of $P < 0.05$. For illustration of the results, we present the ERP of the electrode with the highest significance.(see Supplementary Fig. 1).

Supplementary References

1. Antony, J. W., Gobel, E. W., O'Hare, J. K., Reber, P. J. & Paller, K. a. Cued memory reactivation during sleep influences skill learning. *Nat. Neurosci.* **15,** 1114–6 (2012).

2. Schreiner, T. & Rasch, B. Boosting Vocabulary Learning by Verbal Cueing During Sleep. *Cereb. Cortex* 1–11 (2014).

3. Walsh, M. M. & Anderson, J. R. Learning from experience: Event-related potential correlates of reward processing, neural adaptation, and behavioral choice. *Neuroscience and Biobehavioral Reviews* **36,** 1870–1884 (2012).

4. Miltner, W. H. R., Braun, C. H. & Coles, M. G. H. Event-Related Brain Potentials Following Incorrect Feedback in a Time-Estimation Task: Evidence for a 'Generic' Neural System for Error Detection. *Journal of Cognitive Neuroscience* **9,** 788–798 (1997).
